# Supplementary material for: Multi-modal quantification of pathway activity with MAYA
Source: Nat Commun. 2023 Mar 25;14:1668. doi: 10.1038/s41467-023-37410-2 (PMC10039856; doi:10.1038/s41467-023-37410-2)
Supplement: Supplementary file 1 — Supplementary Information [file 41467_2023_37410_MOESM1_ESM.pdf]

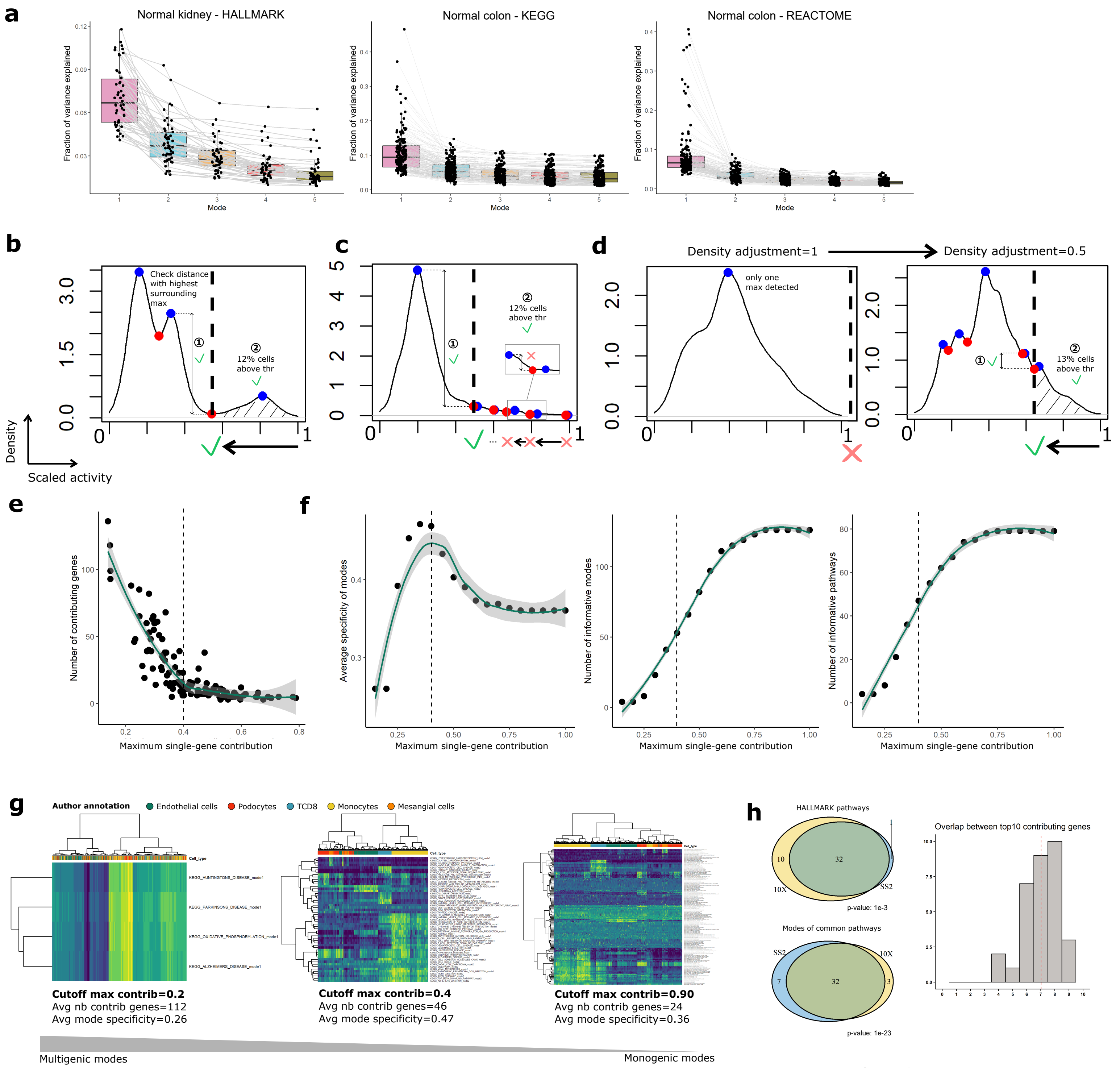

i

Dataset	Number of cells	Gene list	Comput. time Activity matrix	Comput. time Cell annot	Total comput. time
Kidney (subset)	1,252	PanglaoDB Kidney (57 cell types)	3.2 secs	0.19 secs	~ 3 secs
Kidney (pre-filtering)	125,139	PanglaoDB Kidney (57 cell types)	6.1 mins	9.7 mins	~ 15 mins
Kidney (post-filtering)	72,501	PanglaoDB Kidney (57 cell types)	3.1 mins	2.4 mins	~ 5 mins
Colon (subset)	1,415	PanglaoDB GI tract (60 cell types)	2.1 secs	0.12 secs	~ 2 secs
Colon	27,414	PanglaoDB GI tract (60 cell types)	1.0 min	12 secs	~ 1 min
Larynx	5,179	PanglaoDB Basic (47 cell types)	9.9 secs	1.1 secs	~ 11 secs
Ovary	16,815	PanglaoDB Basic (47 cell types)	38 secs	4.6 secs	~ 45 secs

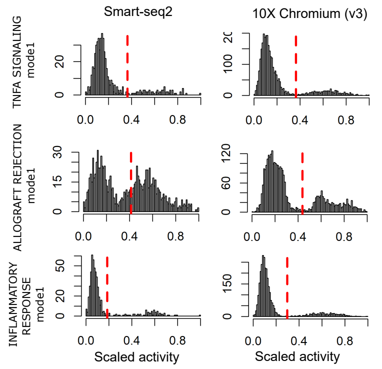

Supplementary Fig.1:

(a) Overlaid jitter and boxplot representations of the fraction of variance explained by PC1 to PC5, for a PCA computed with either HALLMARK pathways on the kidney dataset, and KEGG or REACTOME pathways on the colon dataset; n =50, 186 and 165 independent gene lists for HALLMARK, KEGG and REACTOME respectively; Center line, median; box limits, upper and lower quartiles; whiskers, 1.5× interquartile range. **(b,c)** Examples of density curve of activity scores for one mode of activation. Detected maxima in density are colored in blue and minima in red. MAYA selects a mode as relevant when it has a local density minimum that (i) is low enough compared with surrounding highest maximum and that (ii) splits the datasets into two fractions that are of a minimal size (Methods). Minima are screened in decreasing order on the x-axis and MAYA stops either when a minimum meets the criteria or when it is to the left of the highest density maximum. In (a) the first minimum at the right meets the two criteria and for (b) the fifth. They are marked by a vertical dashed line. **(d)** When no minima are detected with the first density adjustment parameter, a more fitted adjustment is tested. If minima are found, the procedure described in (b,c) is applied. **(e)** Scatterplot representing the number of contributing genes versus the maximum gene contribution, for the first five modes of all pathways from the KEGG pathway list on the kidney dataset. Error band represents the 95% confidence interval for locally estimated scatterplot smoothing (LOESS). **(f)** Scatterplots of the average mode specificity, the number of informative modes and the number of informative pathways according to the maximum single-gene contribution. Default cut-off of maximum single gene variance (0.4) was chosen to maximize the specificity of the modes of activation and is indicated as a vertical dashed line. Error bands represent the 95% confidence interval for locally estimated scatterplot smoothing (LOESS). **(g)** Heatmap of activity matrices for different cut-off of single-gene contribution: 0.2, 0.4 and 0.9. **(h)** Venn diagram displaying respectively the intersection of pathways selected by MAYA and the intersection of modes for common pathways in the PBMC 10X Chromium and Smart-Seq2 datasets. Significance of the overlap is assessed using Fisher's exact test. Barplot representation of the size of the overlap between top10 contributing genes for modes selected in both 10X Chromium and Smart-Seq2 datasets. Examples of distributions of activity scores for three modes in 10X Chromium and Smart-Seq2 datasets. Bimodality thresholds are marked by a vertical dashed line. **(i)** Computing time on different datasets for the two main modules of the function MAYA_predict_cell_type (building activity matrix and annotating cells), using PanglaoDB (44 markers on average per cell type) restricted to cell types expected in the tissue corresponding to the datasets.

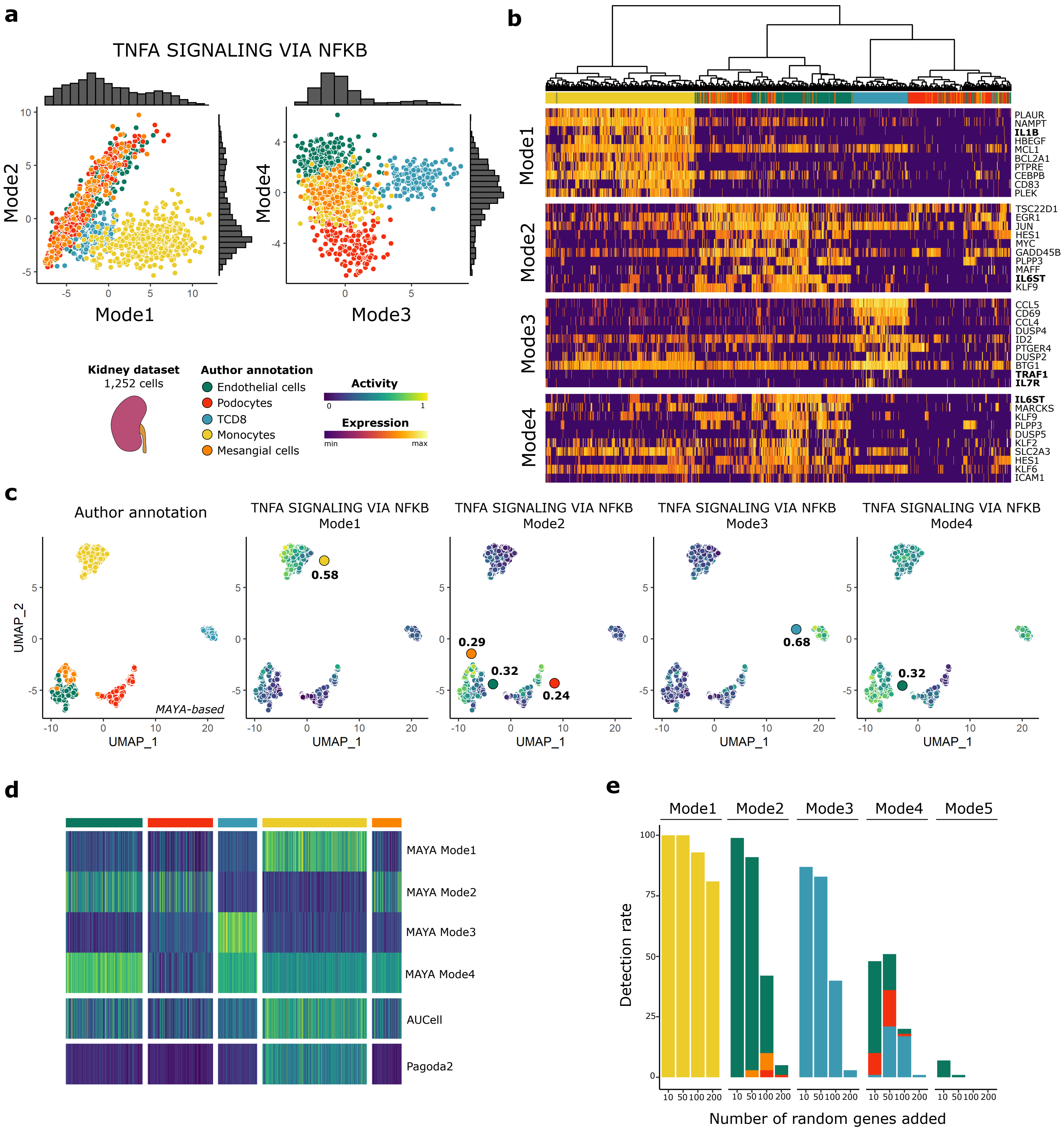

Supplementary Fig.2:

(a) Scatterplot of Mode 2 versus Mode 1 and Mode 4 versus Mode 3 cell activity scores, for the pathway TNFA signaling via NFKB on the kidney dataset. Associated density histograms are indicated on the sides of the graphs. **(b)** Heatmap of scaled gene expression for top10 contributing genes for the four activation modes of TNFA signaling via NFKB pathway, ordered by decreasing contribution. **(c)** UMAP representation of activity matrix of Hallmark pathways, cells are colored according to author annotation, or activity scores of the four modes of TNFA signaling via NFKB pathway. Specificity score of cell populations is displayed next to relevant clusters. **(d)** Heatmap of activity scores computed by Pagoda2, AUCell and MAYA for TNFA signaling via NFKB pathway, cells are grouped according to author annotation. **(e)** Barplot representation of the detection rate of modes 1 to 5 for the pathway TNFA signaling via NFKB when adding various numbers of random genes to the pathway gene list (n=100 experiments each). Barplots are colored according to the cell population with the highest specificity score for the identified mode.

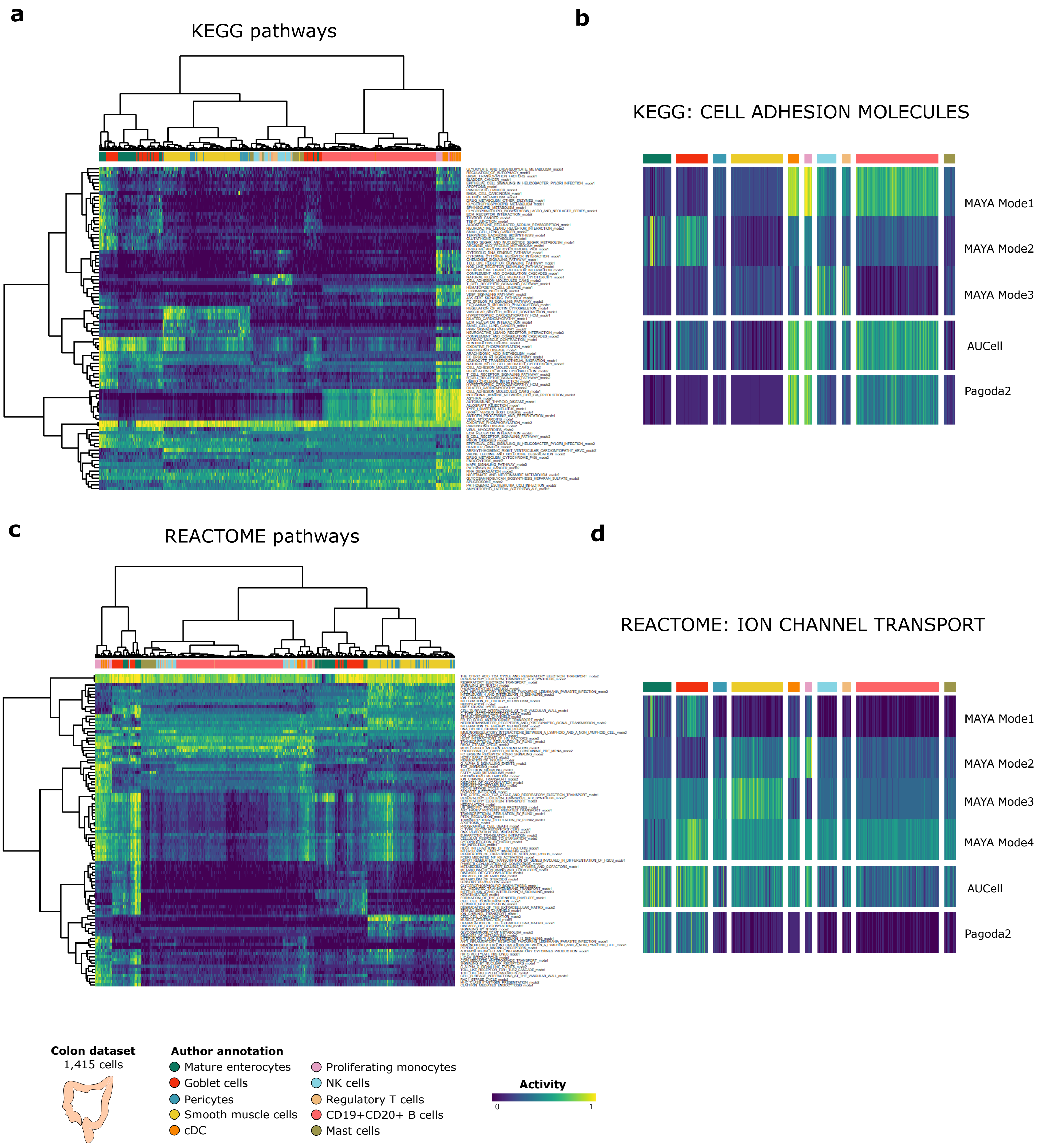

Supplementary Fig.3:

(a) Heatmap of activity matrix computed on colon dataset with MSigDB KEGG pathways, initial author annotation is indicated above heatmap. **(b)** Heatmap of activity scores computed by Pagoda2, AUCell and MAYA for KEGG Cell Adhesion Molecules pathway, cells are grouped according to author annotation. **(c)** Heatmap of activity matrix computed on colon dataset with MSigDB REACTOME pathways, initial author annotation is indicated above heatmap. **(d)** Heatmap of activity scores computed by Pagoda2, AUCell and MAYA for REACTOME Ion Channel Transport pathway, cells are grouped according to author annotation.

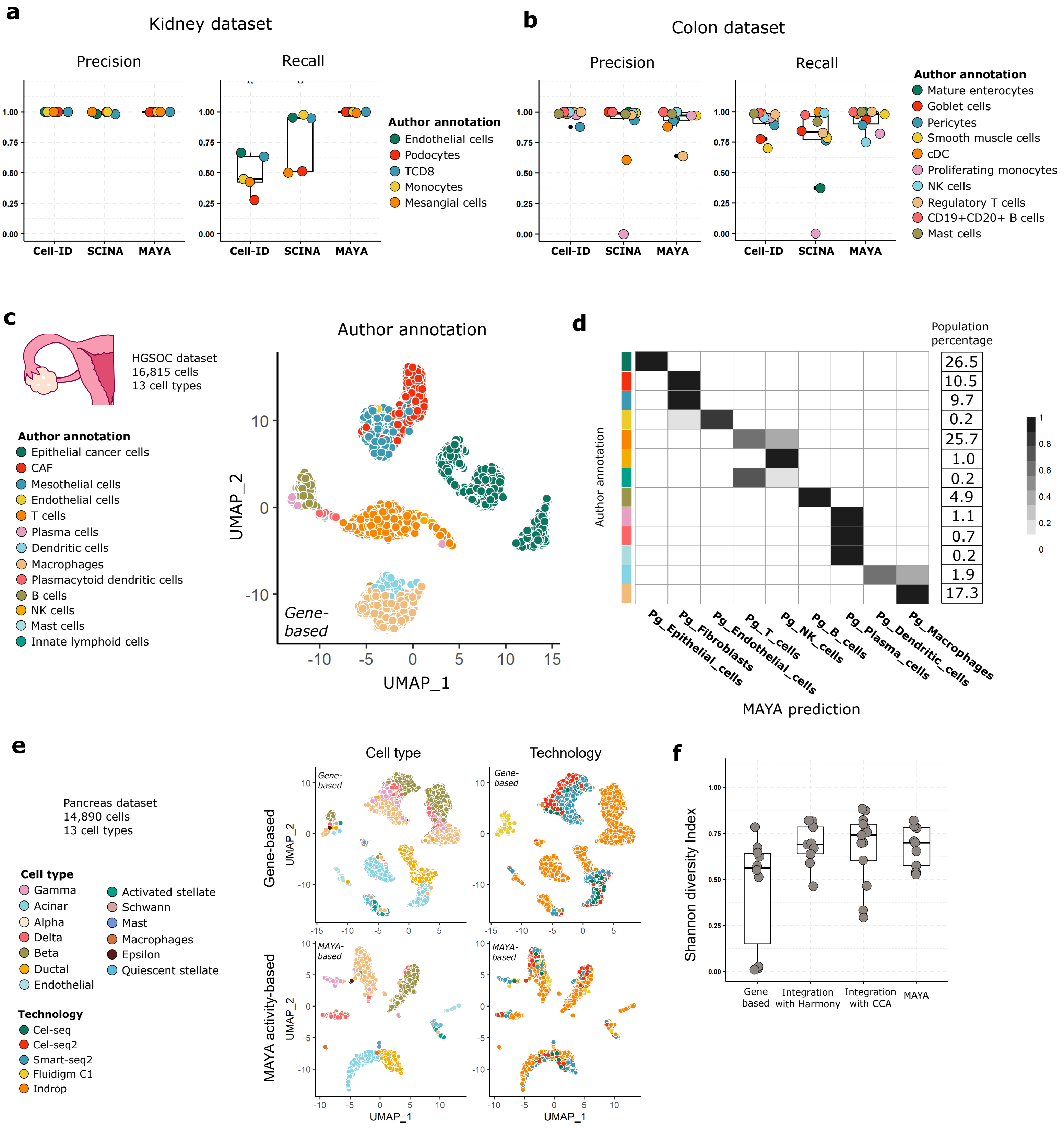

Supplementary Fig.4:

(a,b) Overlaid jitter and boxplot representation of precision and recall for automatic annotation of the kidney and colon datasets using Cell-ID, SCINA and MAYA, datapoints are colored according to author annotation; n = 5 and 10 cell types respectively; Center line, median; box limits, upper and lower quartiles; whiskers, 1.5× interquartile range; Adjusted p-values from two-sided Wilcoxon test are symbolized with: * : <0.05, ** : <0.01, *** : <0.001, **** : <0.0001. Exact p-value for recall in kidney is 0.0097 for both Cell-ID and SCINA versus MAYA. **(c)** Gene-based UMAP representation of the ovary dataset, cells are colored according to author annotation. **(d)** Heatmap representing for each author annotation (rows) the fraction of cells labelled with each MAYA annotation (columns) for the ovary dataset. The proportion of each author annotation in the dataset is indicated on the right side of the heatmap. **(e)** UMAP representation of the pancreas dataset, either gene-based or based on activity matrix of PanglaoDB cell-type markers lists, cells are colored according to cell type or to technology. **(f)** Overlaid jitter and boxplot representation of Shannon Diversity Index (SDI), for clusters derived from gene-based dimensionality reduction, Harmony dimensionality reduction, CCA dimensionality reduction and MAYA activity matrix of the pancreas dataset; n = 10, 9, 13 and 9 independent clusters respectively; Center line, median; box limits, upper and lower quartiles; whiskers, 1.5× interquartile range.

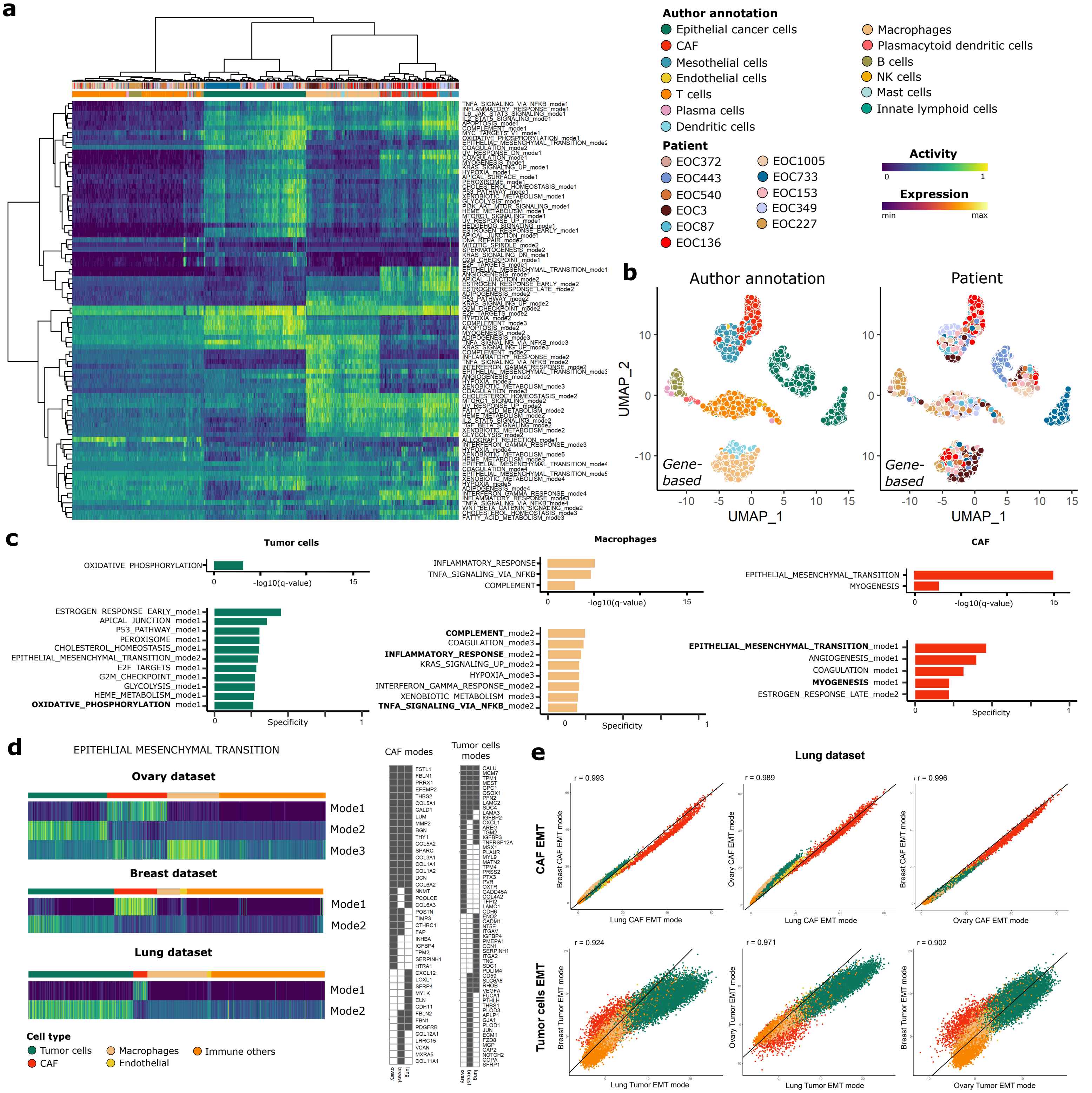

Supplementary Fig.5:

(a) Heatmap of activity matrix computed on ovary dataset with MSigDB Hallmark pathways, initial author annotation is indicated above heatmap. **(b)** Gene-based UMAP representation of expression matrix, cells are colored according to author annotation and patient. **(c)** Barplots displaying the Hallmark pathways significantly enriched using GSEA in over-expressed genes (x-axis corresponds to -log10 Benjamini-Hochberg adjusted p-values, p-values being evaluated using an adaptive multilevel Monte Carlo sampling scheme as implemented in fast GSEA algorithm) and the MAYA top specific modes (x-axis corresponds to specific scores, in bold are indicated the gene lists found with GSEA analysis) for tumor cells, macrophages and CAF. **(d)** Heatmap of activity scores of the different modes of EMT pathway in the ovary, breast and lung datasets, consensus annotation is indicated above heatmap. Heatmap displaying the presence of genes in the top30 contributing genes of the EMT mode specific to CAF (respectively tumor cells) for the three datasets. **(e)** Scatter plots comparing for each cell in the lung dataset the activity of the CAF (top) and tumor cell (bottom) EMT signatures derived from the lung, ovary and breast datasets. The dark straight line represents y=x and Pearson's correlation coefficient is displayed above each plot.

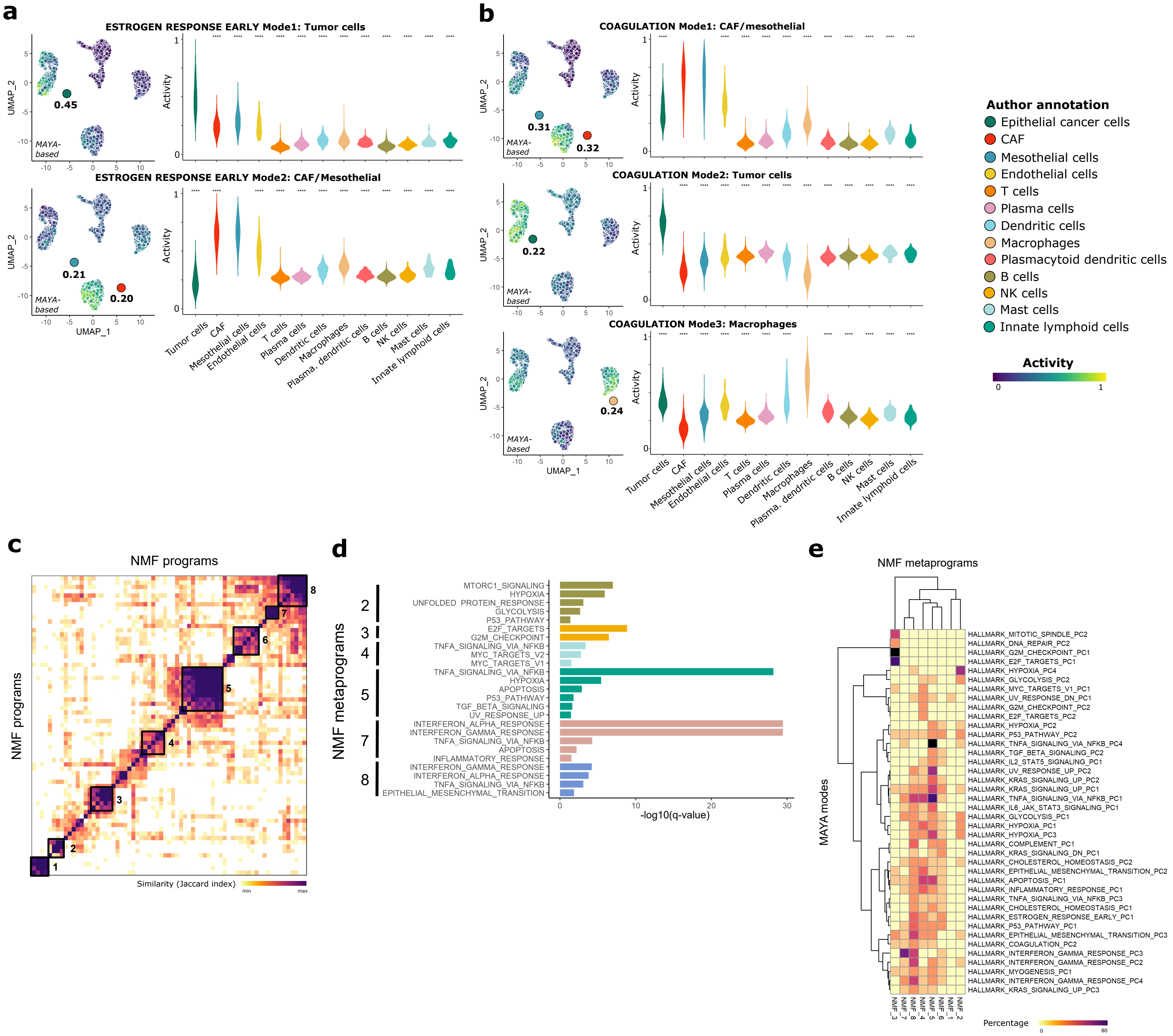

Supplementary Fig.6:

(a,b) UMAP representation of activity matrix of Hallmark pathways, cells are colored according to activity scores of the two Estrogen Response Early modes (respectively three Coagulation modes). Specificity score of cell populations is displayed next to relevant clusters. Violin plots of activity scores for corresponding modes, grouped by author annotation (adjusted p-values from two-sided Wilcoxon test are symbolized with: * : <0.05, ** : <0.01, *** : <0.001, **** : <0.0001). **(c)** Heatmap representation of the similarity of robust NMF programs, found in epithelial ovarian cancer cells for each patient individually. Similarity is assessed using a Jaccard similarity index. Main metaprograms are circled in black. **(d)** Barplot displaying the Hallmark pathways enriched in metaprograms using a hypergeometric test. X-axis corresponds to -log10 Benjamini-Hochberg adjusted p-values. **(e)** Heatmap displaying the percentage of the top20 contributing genes of MAYA modes overlapping each metaprogram. Only MAYA modes with a percentage of overlap superior to 15% with one metaprogram were kept for clarity.

Supplementary methods

Dataset pre-processing

Kidney dataset: Metadata was built by combining table S11 from Young et al. providing a cell manifest with table S2 providing author's cell type annotation. Only protein-coding genes were kept for downstream analysis. Data was provided for 125,139 cells, with 72,502 cells passing the author's quality control criteria. MAYA assisted annotation function was run on the dataset before and after QC filtering to evaluate its scalability to large datasets. For our detailed pathway analysis, only normal kidney cells were selected based on author's annotation (categories "Normal_mature_kidney" and "Normal_mature_kidney_immune"). Cells from 5 distinct cell types out of 28 were selected after default Seurat processing and clustering (aliases 8T, AV2, MNP1, G and M) for a total 1,252 cells.

Colon dataset: Only protein-coding genes were kept for downstream analysis. MAYA assisted annotation function was run on this full dataset - including normal, tumor and border cells - to evaluate its scalability to large datasets. For our detailed pathway analysis, cells from Class "Normal" and from 10 out of the 35 cell types identifies by the authors were selected, representing a total of 1,415 cells.

Ovary dataset: Only cells labelled as treatment-naïve for the treatment phase metadata field were kept for downstream analysis, representing a total of 16,815 cells.

Larynx dataset: The files for the two patients were combined and all 5,179 cells were kept for downstream analyses.

Pancreas dataset: No further preprocessing was required for this dataset; all 14,890 cells were kept for downstream analyses.

Breast dataset: No further preprocessing was required for this dataset; all 16,537 cells were kept for downstream analyses.

Lung dataset: No further preprocessing was required for this dataset; all 32,493 cells were kept for downstream analyses. cells.

PBMC dataset: We separated the downloaded dataset in two distinct objects the data generated using 10X Chromium v3 (10X) and using Smart-Seq2 (SS2), for a total of respectively 2,993 and 526 cells.

Databases pre-processing

PanglaoDB: Markers lists are categorized by organs. Some can be considered as generic organs that should always be tested for a dataset (connective tissue, smooth muscle, immune system, vasculature,

blood, epithelium, skeletal muscle), others are more specific such as kidney or lungs and can be loaded on demand. The full Panglao gene list can be loaded as well. Kidney related lists were loaded for the kidney dataset, GI tract related lists for the colon dataset, and finally no other list than generic types for the larynx and ovary datasets.

MSigDB: For the Reactome database, only pathways comprising between 100 and 300 genes were kept for efficiency purposes, which represents 165 pathways kept over 1615.
