# Supplementary material for: Multi-modal quantification of pathway activity with MAYA
Source: Nat Commun. 2023 Mar 25;14:1668. doi: 10.1038/s41467-023-37410-2 (PMC10039856; doi:10.1038/s41467-023-37410-2)
Supplement: Supplementary file 3 — Reporting Summary [file 41467_2023_37410_MOESM3_ESM.pdf]

nature portfolio

Corresponding author(s): Céline VALLOT

Last updated by author(s): Feb 24, 2023

Reporting Summary

Nature Portfolio wishes to improve the reproducibility of the work that we publish. This form provides structure for consistency and transparency in reporting. For further information on Nature Portfolio policies, see our Editorial Policies and the Editorial Policy Checklist.

Statistics

For all statistical analyses, confirm that the following items are present in the figure legend, table legend, main text, or Methods section.

n/a	Confirmed	
☐	☒	The exact sample size (*n*) for each experimental group/condition, given as a discrete number and unit of measurement
☐	☒	A statement on whether measurements were taken from distinct samples or whether the same sample was measured repeatedly
☐	☒	The statistical test(s) used AND whether they are one- or two-sided<br>*Only common tests should be described solely by name; describe more complex techniques in the Methods section.*
☒	☐	A description of all covariates tested
☒	☐	A description of any assumptions or corrections, such as tests of normality and adjustment for multiple comparisons
☐	☒	A full description of the statistical parameters including central tendency (e.g. means) or other basic estimates (e.g. regression coefficient) AND variation (e.g. standard deviation) or associated estimates of uncertainty (e.g. confidence intervals)
☐	☒	For null hypothesis testing, the test statistic (e.g. *F*, *t*, *r*) with confidence intervals, effect sizes, degrees of freedom and *P* value noted<br>*Give P values as exact values whenever suitable.*
☒	☐	For Bayesian analysis, information on the choice of priors and Markov chain Monte Carlo settings
☒	☐	For hierarchical and complex designs, identification of the appropriate level for tests and full reporting of outcomes
☒	☐	Estimates of effect sizes (e.g. Cohen's *d*, Pearson's *r*), indicating how they were calculated

Our web collection on statistics for biologists contains articles on many of the points above.

Software and code

Policy information about availability of computer code

Data collection	NA
Data analysis	MAYA is available as an R package on GitHub at https://github.com/One-Biosciences/MAYA/. Requires R >= 4.0.5. Code for reproducing data analysis and plots is available at https://github.com/One-Biosciences/MAYA-figures/.<br>Analysis of scRNA-Seq data involved the use of the following public R packages: msigdbr (https://github.com/igordot/msigdbr, v7.5.1), tidyr (https://github.com/tidyverse/tidyr, v1.2.0), Seurat (https://github.com/satijalab/seurat, v4.3.0), SingleCellExperiment (https:// bioconductor.org/packages/release/bioc/html/SingleCellExperiment.html, v1.12.0), SCINA (https://github.com/jcao89757/SCINA, v1.2.0), SeuratData (https://github.com/satijalab/seurat-data, v0.2.2), AUCell (https://github.com/aertslab/AUCell, v1.12.0), pagoda2 (https:// github.com/kharchenkolab/pagoda2, v1.0.10), CellID (https://github.com/RausellLab/CelliD, v0.1.0), harmony (https://github.com/ immunogenomics/harmony, v0.1.0), fgsea (https://github.com/ctlab/fgsea, v1.16.0).

For manuscripts utilizing custom algorithms or software that are central to the research but not yet described in published literature, software must be made available to editors and reviewers. We strongly encourage code deposition in a community repository (e.g. GitHub). See the Nature Portfolio guidelines for submitting code & software for further information.

Data

Policy information about availability of data

All manuscripts must include a data availability statement. This statement should provide the following information, where applicable:

- Accession codes, unique identifiers, or web links for publicly available datasets
- A description of any restrictions on data availability
- For clinical datasets or third party data, please ensure that the statement adheres to our policy

Data availability was described in the manuscript. Public datasets are freely available as follow.
Kidney dataset: DOI 10.1126/science.aat1699 (https://www.science.org/doi/suppl/10.1126/science.aat1699/suppl_file/aat1699_datas1.gz.zip)
Colon dataset: GSE144735 (https://www.ncbi.nlm.nih.gov/geo/query/acc.cgi?acc=GSE144735)
Ovary dataset: GSE165897 (https://www.ncbi.nlm.nih.gov/geo/query/acc.cgi?acc=GSE165897)
Larynx dataset: GSE150321 (https://www.ncbi.nlm.nih.gov/geo/query/acc.cgi?acc=GSE150321)
Pancreas dataset: downloaded using SeuratData panc8 v3.0.2 (https://github.com/satijalab/seurat-data)
PBMC dataset: DOI 10.1038/s41587-020-0465-8, downloaded using SeuratData pbmcsca v3.0.0 (https://github.com/satijalab/seurat-data)
Breast dataset: DOI 10.1038/s41422-020-0355-0 (https://www.dropbox.com/sh/nbx7v3om85wkfoq/AACpeZEZ4RNQwMW37Q7AHxExa?dl=1)
Lung dataset: DOI 10.1038/s41467-020-16164-1 (https://www.dropbox.com/sh/byext689ffg77pj/AACp5jI2RRxndurKn2B0T-VWa?dl=1)

Reference databases:
PanglaoDB: DOI 10.1093/database/baz046 (https://panglaodb.se/markers/PanglaoDB_markers_27_Mar_2020.tsv.gz)
MSigDB gene lists: DOI 10.1073/pnas.0506580102 (https://data.broadinstitute.org/gsea-msigdb/msigdb/release/7.4/h.all.v7.4.symbols.gmt, https://data.broadinstitute.org/gsea-msigdb/msigdb/release/7.4/c2.cp.kegg.v7.4.entrez.gmt, https://data.broadinstitute.org/gsea-msigdb/msigdb/release/7.4/c2.cp.reactome.v7.4.symbols.gmt).

Human research participants

Policy information about studies involving human research participants and Sex and Gender in Research.

Reporting on sex and gender	NA
Population characteristics	NA
Recruitment	NA
Ethics oversight	NA

Note that full information on the approval of the study protocol must also be provided in the manuscript.

Field-specific reporting

Please select the one below that is the best fit for your research. If you are not sure, read the appropriate sections before making your selection.

[x] Life sciences [ ] Behavioural & social sciences [ ] Ecological, evolutionary & environmental sciences

For a reference copy of the document with all sections, see nature.com/documents/nr-reporting-summary-flat.pdf

Life sciences study design

All studies must disclose on these points even when the disclosure is negative.

Sample size	We considered that the number of cells studied for each of the eight scRNA-Seq dataset was sufficient for their analysis (Kidney: 1,252 cells; Colon: 1,415 cells; Ovary: 16,815 cells; Larynx: 5,179 cells; Lung: 32,493 cells; Breast: 16,537 cells; Pancreas: 14,890 cells; PBMC: 3,519 cells).
Data exclusions	We excluded from single-cell analysis cells based on Quality Control metrics defined by authors in initial publications, from which the datasets originate (based on minimal and maximum coverage, percentage of mitochondiral DNA).
Replication	To demonstrate MAYA properties, we used eight independent public datasets. Accuracy of cell type assignment was assessed in three independent datasets (kidney, colon and ovary). Performances in batch effect correction was assessed in two independent datasets (larynx and pancreas). Reproducibility of MAYA modes was assessed in three independent cancer datasets (ovary, lung and breast).
Randomization	NA
Blinding	No blinding was performed, as sample labels are necessary to perform computational analyses.

Reporting for specific materials, systems and methods

We require information from authors about some types of materials, experimental systems and methods used in many studies. Here, indicate whether each material, system or method listed is relevant to your study. If you are not sure if a list item applies to your research, read the appropriate section before selecting a response.

Materials & experimental systems

n/a	Involved in the study
☒	☐ Antibodies
☒	☐ Eukaryotic cell lines
☒	☐ Palaeontology and archaeology
☒	☐ Animals and other organisms
☒	☐ Clinical data
☒	☐ Dual use research of concern

Methods

n/a	Involved in the study
☒	☐ ChIP-seq
☒	☐ Flow cytometry
☒	☐ MRI-based neuroimaging
